# Cerebral thrombophlebitis revealing SARS-CoV-2 infection: About one case

**DOI:** 10.1016/j.amsu.2021.103192

**Published:** 2021-12-21

**Authors:** Ilyass Laaribi, Abdeliliah El Rhalete, Hamza Mimouni, Safaa Kachmar, Safaa Bekkaoui, Amine El Mouhib, Houssam Bkiyar, Brahim Housni

**Affiliations:** a Department of Intensive Care Unit, Mohammed VI University Hospital, Oujda, Morocco; b Faculty of Medicine and Pharmacy, Mohammed First University, Oujda, Morocco; c Mohammed First University Oujda, FMP Oujda, LAMCESM, Oujda, Morocco

**Keywords:** SARS-CoV-2, Cerebral thrombophlebitis, Thromboembolic disease

## Abstract

**Introduction:**

SARS-CoV-2 infection is a pandemic that continues to ravage the world, the list of its complications continues to grow longer every day.

**Case presentation:**

We report the case of a patient admitted to intensive care for cerebral thrombophlebitis revealing a SARS-CoV-2 infection.

**Discussion:**

The inflammatory nature of SARS-CoV-2 infection exposes an increased risk of thrombosis.In this article, we will discuss its mechanism and the anticoagulant treatment modalities.

**Conclusion:**

Besides the typical clinical signs, SARS-CoV-2 infection can manifest as thromboembolic complications such as pulmonary embolism, deep vein thrombosis, and less frequently cerebral thrombophlebitis.

## Highlights

• Several studies have demonstrated an association between SARS-CoV-2 and thromboembolic event.
• These thromboembolic complications may be in the foreground.
• Cerebral thrombophlebitis is a rare and serious complication of SARS-CoV-2 infection.

## 1 Introduction

In December 2019, a new coronavirus responsible of a new disease called SARS-CoV-2 (severe acute respiratory syndrome coronavirus 2) appeared in Wuhan, China. It quickly spread across the world, despite the drastic containment measures put in place by several countries [1,2], the clinical picture of SARS-CoV-2 patients varied from simple symptoms (cough, dyspnea, chest pain, diarrhea, headache, etc.) to organ failure (ARDS, states of shock, heart and kidney damage) [3]. Along with respiratory involvement, the onset of several thromboembolic events has been described during infection, which suggests that this virus has a high thrombotic potential.

Cerebral thrombophlebitis is a rare and potentially serious thromboembolic complication, which may be secondary to the thrombotic state produced by this virus [4].

## 2 Presentation of the clinical case

We report in this work the case of a patient admitted to intensive care unit for a secondary disorder of consciousness cerebral thrombophlebitis revealing a SARS-CoV-2 infection, in whom a PCR test on nasopharyngeal swab and serology were carried out returning positive for the SARS-CoV-2.

The patient was an unvaccinated 42-year-old woman, with no notion of taking oral contraception, with no notable pathological history admitted to the intensive care unit for the management of an impaired consciousness associated with a fever, and progressive worsening dyspnea evolving since 4 days.

The clinical examination on admission demonstrated a Glasgow Coma Score of 13/15, and the neurological assessment revealed a hypotonia of all 4 limbs with depressed osteotendinous reflexes in the lower limbs.

Monitoring found a patient hemodynamically stable, normotensive with arterial pressure 125/65 mmHg, normocardium at 98 bpm, an oxygen saturation level of 96% and a respiratory rate of 14 breaths/min, as well as a fever up to 39°.

An arterial blood gas test revealed a pH of 7.41, PO2: 75 mmHg, PCO2: 36.5 mmHg, oxygen saturation: 96% without oxygen supply.

**Fig. 1:**
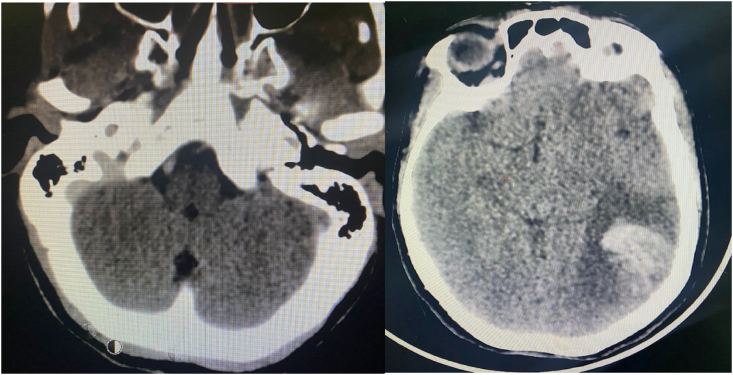
CT image showing left hemorrhagic infarction over left lateral sinus thrombophlebitis.

**Fig. 2:**
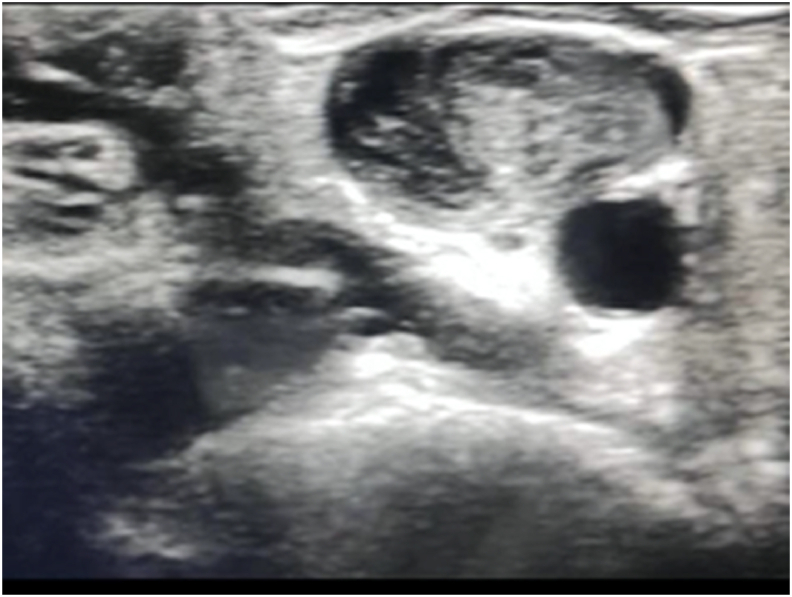
Ultrasound image of left internal jugular vein thrombosis.

**Fig. 3:**
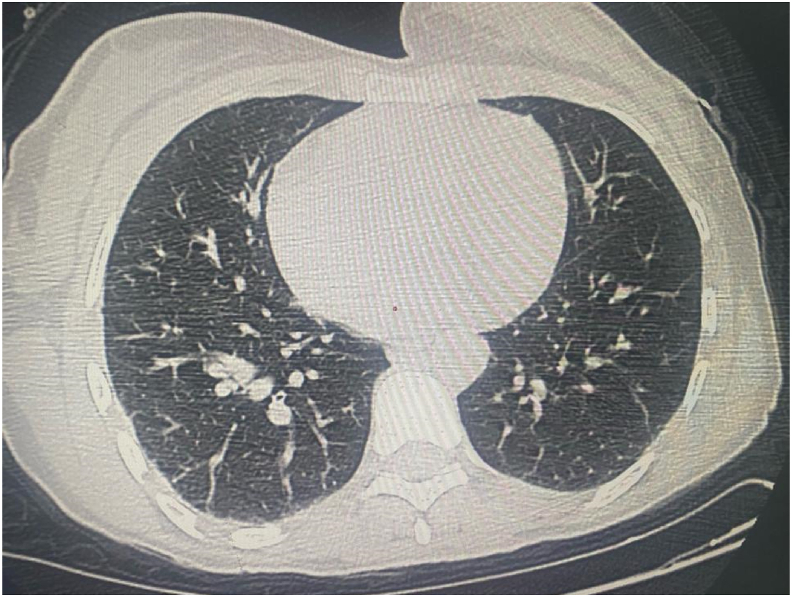
CT image not showing a SARS-CoV-2 type attack.

To explore her disorder of consciousness, a brain CT was performed showing a thrombophlebitis of the left lateral sinus, extended to the origin of the ipsilateral internal jugular vein (Fig. 1), complicated by a left posterior parietal hemorrhagic infarction (Fig. 2)).A chest CT scan was also performed showing no parenchymal involvement or signs of pulmonary embolism (Fig. 3).

A PCR test on a nasopharyngeal sample was positive as well as a positive SARS-CoV-2 serology (positive IgG and IgM).

The biological check-up was in favor of lymphopenia at 760/mm³, hemoglobin at 12.4 g/dl, platelet count at 523000/mm³, hyperleukocytosis at 12000/mm³, a C-reactive protein at 100 mg/l with a negative PCT at 0.05 ng/ml, ferritin at 1000 mcg/L and a dosage of interleukin 6 at 160 pg/l, high D-dimer levels at 6000 μg/l with high fibrinogen at 9 g/l, kidney and liver functions were normal.

Faced with this atypical thromboembolic complication, a thrombophilia assessment was performed returning negative (no deficiency in protein C, protein S and factor V).

Therapeutically, we put the patient on Vitamin C, Zinc, corticosteroid therapy (solumedrol) 120 mg per day, curative anticoagulation with enoxaparin 6000UI/12h, Tocilizumab 600 mg single dose and an anticomitial treatment with sodium valproate 500mg/8h.

The evolution was favorable after 7 days with an improvement in the state of consciousness. We also noticed an improvement in lymphopenia and inflammatory workup. The patient was transferred to the neurology department then she returned home after 2 weeks of hospitalization.

## 3 Discussion

SARS-CoV-2 infection is characterized by an increased risk of thrombosis, which is explained by the elevation of fibrinogen levels. Thrombotic complications were reported in approximately one-third of critically ill patients [9], the increase in pro-inflammatory cytokines including Interleukin 1 and 6 leads to activation of coagulation and the generation of thrombin. This is the case in our patient who had a fibrinogen level at 9 g/l, D-dimers highly superior to the normal value, which could explain this thromboembolic event [5,6].

SARS-CoV-2 enters cells via ACE 2 present in alveolar epithelial cells and then endothelial cells in the kidney of the heart of the intestine. This phenomenon will promote the release of the renin angiotensin aldosterone system, which will lead to platelet adhesion and increase the risk of developing thromboembolic diseases [[7], [8], [9]].

Curative anticoagulation should be started in the event of thromboembolic diseases. Several types of therapy exist: the use of FNH is indicated in critically ill patients with a high risk of bleeding because it can be easily withheld. The use of low molecular weight fractionated heparin at a curative dose is most often indicated in patients hospitalized in intensive care. The choice of anticoagulation depends on kidney function, blood count and risk of bleeding. Curative anticoagulation is indicated for patients with history of thromboembolic disease, Body mass index >30 Kg/m^2^, D-dimer superior than six times the normal value and fibrinogen superior than 8 g/l [8].

## 4 Conclusion

SARS-CoV-2 is characterized by an increased thrombotic risk that can lead to several thromboembolic events including cerebral thrombophlebitis which has a poor prognosis. The fight against the inflammatory nature and the curative anticoagulation in certain indications reduces the risk of occurrence of these thromboembolic complications.

This work is reported in line with the 2020 SCARE guidelines [10].

## Provenance and peer review

Not commissioned, externally peer reviewed.

## Sources of funding

This research was not funded.

## Ethical approval

This is a case report that does not require a formal ethical committee approval. Data were anonymously registered in our database. Access to data was approved by the head of the department.

## Consent

Written informed consent was obtained from the patient for publication of this case report and accompanying images. A copy of the written consent is available for review by the Editor-in-Chief of this journal on request.

## Author contribution

Dr Ilyass Laaribi and Dr. Abdelilah El Rhalete: are principal investigators that collected and analyzed data, wrote the manuscript and prepared the final draft for the submission.

Prof. Brahim Housni and Prof. Houssam Bkiyar: supervised the research project and approved the final draft for publication.

All authors approved the final version of the manuscript.

## Registration of Research Studies

This is not an interventional study. We only reported the patient's findings from our database as a case series.

## Guarantor

Dr Ilyass Laaribi and Dr Abdelilah El Rhalete.

## Declaration of competing interest

The authors declare no conflict of interest.
